# The burden of child maltreatment in China: a systematic review

**DOI:** 10.2471/BLT.14.140970

**Published:** 2015-01-20

**Authors:** Xiangming Fang, Deborah A Fry, Kai Ji, David Finkelhor, Jingqi Chen, Patricia Lannen, Michael P Dunne

**Affiliations:** a Department of Applied Economics, College of Economics and Management, China Agricultural University, No. 17 Qinghuadong Road, Haidian District, Beijing, 100083, China.; b Moray House School of Education, University of Edinburgh, Edinburgh, Scotland.; c Crimes Against Children Research Center, University of New Hampshire, Durham, United States of America.; d Institute of Child and Adolescent Health, Peking University Health Science Center, Beijing, China.; e Child Protection Program, UBS Optimus Foundation, Zurich, Switzerland.; f The Children and Youth Research Centre, Queensland University of Technology, Kelvin Grove, Australia.

## Abstract

**Objective:**

To estimate the health and economic burdens of child maltreatment in China.

**Methods:**

We did a systematic review for studies on child maltreatment in China using PubMed, Embase, PsycInfo, CINAHL-EBSCO, ERIC and the Chinese National Knowledge Infrastructure databases. We did meta-analyses of studies that met inclusion criteria to estimate the prevalence of child neglect and child physical, emotional and sexual abuse. We used data from the 2010 global burden of disease estimates to calculate disability-adjusted life-years (DALYs) lost as a result of child maltreatment.

**Findings:**

From 68 studies we estimated that 26.6% of children under 18 years of age have suffered physical abuse, 19.6% emotional abuse, 8.7% sexual abuse and 26.0% neglect. We estimate that emotional abuse in childhood accounts for 26.3% of the DALYs lost because of mental disorders and 18.0% of those lost because of self-harm. Physical abuse in childhood accounts for 12.2% of DALYs lost because of depression, 17.0% of those lost to anxiety, 20.7% of those lost to problem drinking, 18.8% of those lost to illicit drug use and 18.3% of those lost to self-harm. The consequences of physical abuse of children costs China an estimated 0.84% of its gross domestic product – i.e. 50 billion United States dollars – in 2010. The corresponding losses attributable to emotional and sexual abuse in childhood were 0.47% and 0.39% of the gross domestic product, respectively.

**Conclusion:**

In China, child maltreatment is common and associated with large economic losses because many maltreated children suffer substantial psychological distress and might adopt behaviours that increase their risk of chronic disease.

## Résumé

**Objectif:**

Estimer la charge sanitaire et économique de la maltraitance des enfants en Chine.

**Méthodes:**

Nous avons réalisé une revue systématique des études sur la maltraitance des enfants en Chine en utilisant les bases de données PubMed, Embase, PsycInfo, CINAHL-EBSCO, ERIC et de l'Infrastructure nationale de connaissances chinoises. Nous avons effectué les méta-analyses des études qui satisfaisaient les critères d'inclusion, afin d'estimer la prévalence de l'abandon moral des enfants et des abus physiques, psychologiques et sexuels des enfants. Nous avons utilisé les données des estimations 2010 de la charge mondiale de morbidité pour calculer l'espérance de vie corrigée de l'incapacité (EVCI) perdue du fait de la maltraitance des enfants.

**Résultats:**

À partir de 68 études, nous avons estimé que 26,6% des enfants de moins de 18 ans ont souffert d'abus physiques, 19,6% d'abus psychologiques, 8,7% d'abus sexuels et 26,0% d'abandon moral. Nous estimons que les abus psychologiques représentent 26,3% de l'EVCI perdue à cause des troubles mentaux et 18,0% de l'EVCI perdue à cause des comportements autodestructeurs. Les abus physiques pendant l'enfance représentent 12,2% de l'EVCI perdue à cause de la dépression, 17,0% à cause de l'anxiété, 20,7% à cause des problèmes de surconsommation d'alcool, 18,8% à cause de l'usage des drogues illicites et 18,3% à cause des comportements autodestructeurs. Nous estimons que les conséquences des abus physiques sur les enfants ont coûté à la chine 0,84% de son produit intérieur brut, soit 50 milliards de dollars en 2010. Les pertes correspondantes attribuables aux abus psychologiques et sexuels pendant l'enfance s'élevaient à 0,47% et 0,39% du produit intérieur brut, respectivement.

**Conclusion:**

En Chine, la maltraitance des enfants est courante et associée à des pertes économiques considérables, car de nombreux enfants maltraités souffrent de détresse psychologique profonde et qu'ils peuvent adopter des comportements augmentant leur risque de maladie chronique.

## Resumen

**Objetivo:**

Estimar la carga sanitaria y económica del maltrato infantil en China.

**Métodos:**

Se realizó una revisión sistemática de los estudios sobre el maltrato infantil en China utilizando las bases de datos PubMed, Embase, PsycInfo, CINAHL-EBSCO, ERIC y Chinese National Knowledge Infrastructure. También se efectuó un metaanálisis de los estudios que cumplieron los criterios de inclusión para estimar la prevalencia del abandono infantil y del abuso infantil físico, emocional y sexual. Se emplearon datos de las estimaciones de la carga mundial de morbilidad del año 2010 para calcular los años de vida ajustados por discapacidad (AVAD) perdidos como consecuencia del maltrato infantil.

**Resultados:**

A partir de 68 estudios se estimó que el 26,6 % de los niños menores de 18 años de edad han sufrido abuso físico, el 19,6 % abuso emocional, el 8,7 % abuso sexual y el 26,0 % abandono. Asimismo, se estimó que el abuso emocional en la infancia representa el 26,3 % de los AVAD perdidos a causa de trastornos mentales y el 18,0 % de los perdidos por autolesión. El abuso físico en la infancia representa el 12,2 % de los AVAD perdidos a causa de la depresión, el 17,0 % por ansiedad, el 20,7 % por problemas con el alcohol, el 18,8 % por uso de drogas ilícitas y el 18,3 % por autolesión. Las consecuencias del abuso físico de niños le cuestan a China aproximadamente el 0,84 % de su producto interior bruto, es decir, 50 mil millones de dólares de los Estados Unidos, en 2010. Las pérdidas correspondientes atribuibles al abuso emocional y sexual en la infancia representaron el 0,47 % y el 0,39 % del producto interior bruto, respectivamente.

**Conclusión:**

En China, el maltrato infantil es común y está asociado con grandes pérdidas económicas, ya que muchos niños maltratados sufren trastornos psicológicos importantes y podrían adoptar conductas que aumentan su riesgo de sufrir enfermedades crónicas.

## ملخص

**الغرض:**

تقدير الأعباء الصحية والاقتصادية لإساءة معاملة الأطفال في الصين.

**الطريقة:**

أجرينا استعراضاً منهجياً للدراسات المعنية بإساءة معاملة الأطفال في الصين باستخدام قواعد بيانات PubMed وEmbase وPsycInfo وCINAHL-EBSCO وERIC والبنية الأساسية المعرفية الوطنية الصينية. وأجرينا تحليلات أساسية للدراسات التي لبت معايير الإدراج بغية تقدير معدل انتشار إهمال الأطفال والإيذاء البدني والنفسي والجنسي للأطفال. واستخدمنا البيانات المستمدة من تقديرات العبء العالمي للمرض لعام 2010 لحساب سنوات العمر المصححة باحتساب مدد العجز الضائعة نتيجة لإساءة معاملة الأطفال.

**النتائج:**

أشارت تقديراتنا التي استمدت من 68 دراسة أن 26.6 % من الأطفال دون سن 18 سنة عانوا من الإيذاء البدني و19.6 % من الأطفال دون سن 18 سنة عانوا من الإيذاء النفسي و8.7 % من الأطفال دون سن 18 سنة عانوا من الإيذاء الجنسي و26.0 % من الإهمال. ووفق تقديرنا، يمثل الإيذاء النفسي في الطفولة 26.3 % من سنوات العمر المصححة باحتساب مدد العجز الضائعة بسبب الاضطرابات النفسية وأن 18.0 % منها يرجع إلى إيذاء النفس. ويمثل الإيذاء البدني في الطفولة 12.2 % من سنوات العمر المصححة باحتساب مدد العجز الضائعة بسبب الاكتئاب و17.0 % بسبب القلق و20.7 % بسبب تعاطي الكحوليات الذي يمثل مشكلة و18.8 % بسبب تعاطي المخدرات غير المشروعة و18.3 % بسبب إيذاء النفس. ووفق التقديرات، بلغت التكاليف التي تكبدتها الصين من عواقب الإيذاء البدني للأطفال 0.84 % من ناتجها المحلي الإجمالي - أي 50 مليار دولار أمريكي - في عام 2010. وبلغت الخسائر المقابلة التي تعزى إلى الإيذاء النفسي والجنسي في الطفولة 0.47 % و0.39 % من الناتج المحلي الإجمالي، على التوالي.

**الاستنتاج:**

تشيع في الصين إساءة معاملة الأطفال وترتبط إساءة معاملة الأطفال بخسائر اقتصادية كبيرة نظراً لمعاناة العديد من الأطفال الذين تعرضوا لإساءة المعاملة من ضائقة نفسية شديدة، وقد يسلكوا سلوكيات تزيد من خطورة إصابتهم بأمراض مزمنة.

## 摘要

**目的:**

估计中国儿童虐待的健康和经济负担。

**方法:**

我们使用PubMed、Embase、PsycInfo、CINAHL-EBSCO、ERIC和中国国家知识基础设施数据库（中国知网）对中国儿童虐待研究做了系统回顾。我们对符合纳入标准的研究进行荟萃分析，估计儿童忽视和儿童身体、情感以及性虐待流行率。我们使用2010年全球疾病负担估计的数据计算因儿童虐待导致的伤残调整寿命（DALY）损失。

**结果:**

在我们估计的68份研究中，18岁以下儿童有26.6%遭受身体虐待、19.6%遭受情感虐待、8.7%遭受性虐待、26.0%遭受忽视。据我们估计，童年时期遭受情感虐待分别是26.3%因精神疾病和18.0%因自残导致的DALY损失的根源。而童年时期遭受身体虐待分别是12.2%因抑郁、17.0%因焦虑、20.7%因酗酒、18.8%因使用毒品以及18.3%因自残导致的DALY损失的根源。2010年儿童身体虐待后果令中国国内生产总值（GDP）损失0.84%（即500亿美元）。因童年情感和性虐待造成的损失分别是国内生产总值的0.47%和0.39%。

**结论:**

中国的儿童虐待情况很常见，并伴有巨大的经济损失，这种损失是因为许多受虐儿童遭受巨大的心理压力，可能做出增加慢性病风险的行为。

## Резюме

**Цель:**

Оценить влияние бремени жестокого обращения с детьми в Китае на состояние здоровья и экономику.

**Методы:**

С помощью сведений, полученных из баз данных PubMed, Embase, PsycInfo, CINAHL-EBSCO, ERIC и Китайской национальной инфраструктуры знаний (CNKI), был проведен систематический обзор исследований, посвященных жестокому обращению с детьми в Китае. Для оценки распространения безнадзорности детей, физического, морального и сексуального насилия над детьми был проведен мета-анализ исследований, отвечающих критериям включения в данный обзор. Для расчета количества лет жизни с поправкой на инвалидность (DALY), потерянных в результате жестокого обращения с детьми, были использованы данные оценок глобального бремени болезней за 2010 г..

**Результаты:**

По данным 68 исследований было установлено, что 26,6% детей в возрасте до 18 лет пострадали от физического насилия, 19,6% – от эмоционального насилия, 8,7% – от сексуального насилия и 26,0% – от безнадзорности. Было установлено, что на эмоциональное насилие в детстве приходится 26,3% DALY, потерянных в результате психических расстройств и 18,0% DALY, потерянных в результате нанесения себе увечий. На физическое насилие в детстве приходится 12,2% DALY, потерянных в результате депрессии, 17,0% – из-за страха, 20,7% – из-за пьянства на почве желания уйти от ситуационного стресса, 18,8% – из-за использования запрещенных наркотиков и 18,3% – из-за нанесения себе увечий. Последствия физического насилия над детьми обошлись Китаю предположительно в 0,84% от валового внутреннего продукта, т. е. составили 50 миллиардов долларов США в 2010 году. Убытки, связанные с эмоциональным и сексуальным насилием в детстве, составляли 0,47% и 0,39% от валового внутреннего продукта соответственно.

**Вывод:**

Жестокое обращение с детьми в Китае является распространенным явлением, которое приводит к значительным экономическим убыткам, поскольку многие дети, подвергающиеся жестокому обращению, страдают устойчивыми психологическими расстройствами и могут демонстрировать формы поведения, которые повышают риск возникновения хронических заболеваний.

## Introduction

In the past decade there has been considerable growth in the analysis of the occurrence and consequences of maltreatment and other adversities in childhood.^1^^–^^3^ The maltreatment of children has been found to impair the current and future health and well-being of the children in every country and cultural context in which it has been investigated. The morbidity, disability and mortality caused by child abuse and neglect lead to substantial human suffering, social disadvantage and economic loss.^4^^,^^5^

In China, research in this field has a short history.^6^ There have been no national assessments of child maltreatment and only a few comprehensive provincial studies. However, the results of early descriptive surveys of child sexual^7^^–^^10^ and physical abuse^11^ and some more recent relevant data^12^^,^^13^ have been included in global and regional reviews.^2^^,^^3^^,^^14^^,^^15^ There has also been one systematic review that focused solely on the prevalence of child sexual abuse in China.^16^ There have been no comprehensive studies in China that cover all forms of child maltreatment, examine the consistency of the apparent impacts of such maltreatment on health and well-being or estimate the probable economic consequences. The paucity of official statistics on the incidence of child maltreatment reported to judicial, educational, health and social services – and on the economic costs incurred by such services as a consequence of such maltreatment – also poses a major barrier to the development of an effective and evidence-based policy for child protection in China.

The purpose of this paper was to synthesize the results of previous community-based research on child maltreatment in China. We derived summative estimates of prevalence of emotional, physical and sexual abuse and neglect of children under 18 years of age. We also calculated the magnitude of associations between child maltreatment and consequent poor mental health and health-risk behaviours. We then estimated economic impact of child maltreatment in China. Our observations indicate both the extent to which this major cause of morbidity and disability has been overlooked in China and the research that is still required.

## Methods

### Systematic review

We searched PubMed, Embase, PsycInfo, CINAHL-EBSCO, ERIC and the Chinese National Knowledge Infrastructure for papers published from the inception of each database to 31 December 2013 using search term combinations of *China* with *child abuse*, *emotional abuse*, *physical abuse*, *sexual abuse* or *child neglect* – and their Chinese equivalents. Languages were restricted to English and Chinese. Two reviewers identified and screened potentially relevant articles in Chinese and English and independently assessed the quality of each study that met the inclusion criteria. To identify additional relevant studies, we contacted 18 researchers and organizations involved in child protection in China and checked the reference lists of key narrative reviews on child maltreatment in or around China.^6^^,^^13^^,^^14^^,^^16^^,^^17^

Prevalence studies were included if they met the following criteria: (i) published in a peer-reviewed journal; (ii) participants recruited from a student or general population; (iii) quantitative methods were used to estimate the prevalence of the maltreatment of participants when they were younger than 18 years; (iv) reported a lifetime prevalence of child maltreatment; and (v) the recorded maltreatment had been reported directly by the victims. Studies on the possible consequences – to the victims – of child maltreatment were included if these: (i) represented primary research that had explored the relationship between at least one form of child maltreatment and its impact on employment, education, mental health, physical health, health behaviours, aggression, violence, criminality, exposure to further violence or use of health services;^1^ (ii) included the calculation of odds ratios (ORs) or relative risks (RRs) disaggregated by the type of maltreatment; and (iii) had not sampled on the basis of the presence of any specified outcome – since this would have invalidated the calculation of an OR or RR for that outcome.^18^

The abstract of each article of potential interest was screened to see if the article met our inclusion criteria. We then read the full text of each included article and extracted key variables related to study design and findings. The authors of the articles were contacted if additional information was needed.

Each article was reviewed for data quality by using the Newcastle–Ottawa Scale for case–control and cohort studies^19^ and Boyle’s guidelines for evaluating prevalence studies.^20^ The risk of bias in each included study was determined as in an earlier regional systematic review on child maltreatment.^14^

### Meta-analyses

Following the example of Andrews et al.,^18^ we conducted multiple linear regression analyses to examine the characteristics of the methods that may have influenced previous estimates of the prevalence of child maltreatment. The characteristics examined included type of sample, sample site and size, response type and rate, whether maltreatment was defined as a single or repeated act, whether validated instruments were used and whether specific behavioural questions were asked.

Based on the multiple regression analyses, the unstandardized regression coefficients for the significant predictors of child neglect, emotional abuse and physical abuse were used to adjust the corresponding prevalence. That is, the prevalence of emotional abuse was adjusted from any to repeated abuse, the prevalence of child physical abuse was adjusted to rates generated by validated instruments such as the Conflict Tactics Scale, and the prevalence of child neglect was adjusted to rates reported by studies that had used large samples and asked specific questions about neglect. The prevalence of contact sexual abuse was used as the estimate of the prevalence of any sexual abuse – because the use of any broad definition of non-contact sexual abuse may easily lead to an overestimate of the prevalence of sexual abuse.^21^ As girls are generally more likely to suffer sexual abuse than boys,^3^ we made separate estimates of the prevalence of contact sexual abuse in childhood for females and males.

In seven studies, subtraction of the unstandardized coefficients from the reported prevalence produced negative values.^22^^–^^28^ These studies were excluded from the final meta-analyses. 

Finally, for each of the four types of child maltreatment, a set of adjusted prevalence estimates were combined using random-effects meta-analysis. The separate rates for sexual abuse of boys and girls were combined to produce an overall rate for such abuse – assuming that the Chinese population had 106 males for every 100 females.^29^ The Cochran's Q tests were conducted to assess the heterogeneity across studies.

### Population attributable fractions

To calculate a population attributable fraction, it is necessary to know the prevalence of a risk factor – e.g. maltreatment in childhood – and the RR for the disease or outcome of interest – e.g. depression – given exposure to that risk factor. Since we found only a few articles that reported the effects of child maltreatment on physical health, we focused on outcomes associated with mental health and health-risk behaviours. To match the outcomes with the available global burden of disease categories,^30^ the outcomes were further limited to: current smoker, problem drinking, illicit drug use, self-harm and mental disorder – including depression and anxiety. For each of these outcomes, we attempted to calculate a population attributable fraction for each type of child maltreatment that we considered.

If only the unadjusted ORs for a study were available, we produced corresponding estimates of adjusted ORs using the ratios between adjusted and unadjusted ORs reported for other studies.^18^ Similarly, as only ORs for suicide attempt – rather than self-harm – following sexual abuse were available, we produced estimates of the corresponding OR for self-harm by using the ratio between the ORs for self-harm and suicide attempt following physical abuse. As most studies included in the systematic review reported ORs but not RRs, RRs had to be estimated from the ORs.^31^

In some of our included studies, only RRs for various levels of exposure to a type of maltreatment were available. For these studies, we estimated general RRs for a type of maltreatment by calculating weighted averages – with the numbers of cases at each level of exposure used as the weights.

Finally, for each type of child maltreatment, the estimated RRs were grouped according to outcomes and then combined using random-effects meta-analysis.^32^

### Economic burden

We attempted to estimate the economic losses associated with child maltreatment in China. Following the work of the World Health Organization (WHO)^33^ and Brown,^34^ we estimated the disability-adjusted life-years (DALYs) lost – because of mental health disorders attributable to child maltreatment and health-risk behaviours – and then estimated the monetary value of those DALYs.

For each of the main types of child maltreatment that we considered, a population attributable fraction for an outcome of interest was multiplied by the estimate of the number of DALYs expected to be lost because of that outcome. Population attributable fractions of our selected health and behavioural outcomes (mental disorder, depression, anxiety, current smoker, problem drinking, illicit drug use, and self-harm) were matched to definitions of “mental disorder”, “unipolar depressive disorders”, “anxiety disorders”, “tobacco smoking”, “alcohol use”, “illicit drug use”, and “self-harm” respectively, from the 2010 global burden of disease China study.^30^

For physical abuse and also for emotional abuse, the population attributable fraction for the overall measure of mental disorders was available (Table 1). This was multiplied by an overall estimate of the DALYs lost because of any form of mental ill health. It was often impossible to compute values for individual mental health conditions since population attributable fractions for many such conditions have yet to be estimated. For sexual abuse, population attributable fractions for depression and anxiety – but not for the overall measure of mental ill health – were available (Table 1), and therefore these two individual conditions were used to estimate the DALYs lost because of mental health disorders following sexual abuse in childhood.

**Table 1:** Population attributable fractions and relative risks for health outcomes associated with child maltreatment, China

Type of maltreatment^a^	Mental disorder			Depression			Anxiety			Current smoker			Problem drinking			Illicit drug use			Self-harm	
	RR^b^	PAF, %		RR	PAF, %		RR	PAF, %		RR	PAF, %		RR	PAF, %		RR	PAF, %		RR	PAF, %
Physical abuse	1.87	18.8		1.52	12.2		1.77	17.0		1.40	9.6		1.98	20.7		1.87	18.8		1.84	18.3
Emotional abuse	2.82	26.3		NA	NA		NA	NA		NA	NA		NA	NA		NA	NA		1.84	14.1
Sexual abuse	NA	NA		1.66	5.4		1.53	4.4		2.08	8.6		2.07	8.5		NA	NA		2.39	10.8

NA: not available; PAF: population attributable fraction; RR: relative risk.

^a^ No relevant data were available for child neglect.

^b^ Studies contributing to the RR calculations for each outcome are indicated in Table 2.

As in previous studies,^33^^,^^34^ we assumed that, in monetary terms, one DALY in China was equal to the per-capita gross domestic product. Data on the size of China’s population and its per-capita gross domestic product in 2010 were obtained from the World Bank.^35^

## Results

**Fig. 1:**
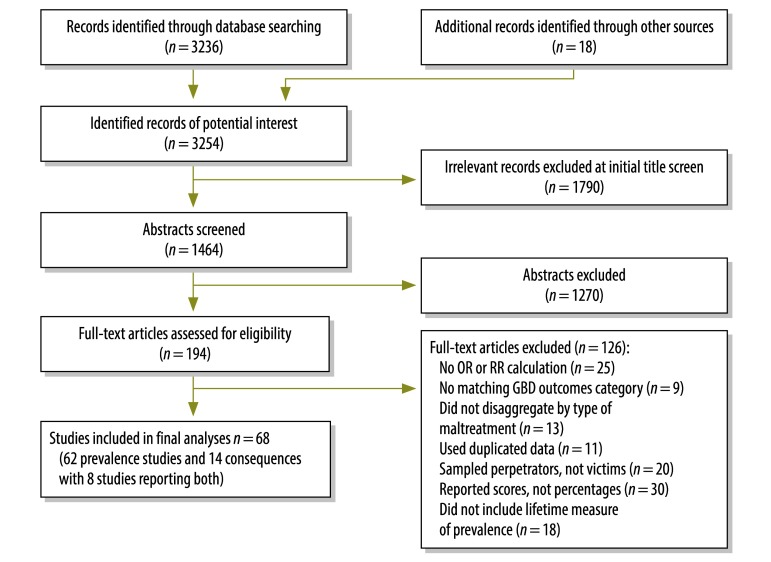
Flowchart for the selection of studies included in the systematic review on child maltreatment in China

GBD: Global Burden of Disease Study; OR: odds ratio; RR: relative risk.

The systematic review identified 68 studies that met our inclusion criteria (Fig. 1), of which 62 reported prevalence estimates and 14 reported consequences. Eight studies were reporting both. For our prevalence estimates, we originally used data from 31 studies on child emotional abuse,^23^^,^^24^^,^^26^^–^^28^^,^^36^^–^^61^ 36 studies on physical abuse,^11^^,^^22^^–^^26^^,^^28^^,^^36^^–^^44^^,^^46^^,^^47^^,^^51^^–^^53^^,^^57^^,^^59^^–^^72^ 18 studies on neglect^23^^,^^27^^,^^28^^,^^36^^,^^43^^,^^44^^,^^46^^,^^47^^,^^49^^,^^50^^,^^53^^–^^56^^,^^59^^,^^73^^–^^75^ and 16 studies on sexual abuse among females^7^^–^^10^^,^^25^^,^^63^^,^^68^^,^^76^^–^^84^ and 12 studies of sexual abuse among males.^9^^,^^10^^,^^63^^,^^68^^,^^76^^,^^78^^–^^83^^,^^85^ (Table 2; available from: http://www.who.int/bulletin/volumes/93/3/14-140970). All of our included studies had a low or medium risk of bias. A weak sampling design, lack of statistical reporting – e.g. a lack of confidence intervals (CIs) – or the use of researcher-developed questions led to a medium risk of bias.

**Table 2:** Studies included for the analysis of child maltreatment in China

Study	Type of study	Maltreatment	Sample size	Risk of bias	Excluded maltreatment in meta-analysis	RR estimation, maltreatment – outcome
Cai (2008)^46^	Prevalence	Emotional, physical, neglect	270	Low	–	–
Chan & Yan (2013)^68^	Prevalence	Physical, sexual	18 341	Low	–	–
Chang & Wang (2008)^56^	Prevalence	Emotional, neglect	230	Medium	–	–
Chen & Dunne (2003)^84^	Prevalence	Sexual	323	Low	–	–
Chen & Liao (2011)^48^	Prevalence	Emotional	430	Medium	–	–
Chen & Liao (2005)^45^	Prevalence	Emotional	484	Medium	–	–
Chen & Liao (2005)^71^	Prevalence	Physical	484	Medium	–	–
Chen et al. (2003)^85^	Prevalence	Sexual	239	Low	–	–
Chen et al. (2006)^65^	Prevalence and consequences	Physical	528	Medium	–	Physical – current smoker
						Physical – problem drinking
Chen et al. (2006)^79^	Prevalence	Sexual	528	Low	–	–
Chen et al. (2010)^80^	Prevalence	Sexual	2 508	Low	–	–
Chen (2004)^83^	Prevalence	Sexual	565	Medium	–	–
Chen (2005)^61^	Prevalence	Emotional, physical	291	Medium	–	–
Chen et al. (2004)^9^	Prevalence and consequences	Sexual	2 300	Low	–	Sexual – current smoker
						Sexual – problem drinking
Chen et al. (2006)^8^	Prevalence and consequences	Sexual	351	Low	–	Sexual – current smoker
						Sexual – problem drinking
Chen et al. (2002)^7^	Prevalence	Sexual	985	Medium	–	–
Chen et al. (2004)^77^	Prevalence	Sexual	892	Medium	–	–
Chen et al. (2008)^26^	Prevalence	Emotional, physical	528	Medium	Emotional	–
Cheng et al. (2010)^75^	Prevalence	Neglect	3 155	Low	–	–
Cheng et al. (2011)^86^	Consequences	Physical	1 628	Low	–	Physical – current smoker
						Physical – problem drinking
						Physical – illicit drug use
Cong et al. (2012)^87^	Consequences	Sexual	4 567	Low	–	Sexual – depression
Chou et al. (2011)^66^	Prevalence	Physical	1 966	Low	–	–
Ding et al. (2007)^37^	Prevalence	Emotional, physical	485	Medium	–	–
Dong et al. (2010)^55^	Prevalence	Emotional, neglect	1 193	Low	–	–
Fuh et al. (2010)^22^	Prevalence	Physical	4 259	Medium	Physical	–
Gao et al. (2011)^43^	Prevalence	Emotional, physical, neglect	301	Medium	–	–
Gao et al. (2013)^73^	Prevalence	Neglect	685	Low	–	–
Gu et al. (2005)^78^	Prevalence	Sexual	1 635	Medium	–	–
Hester et al. (2009)^67^	Prevalence	Physical	498	Medium	–	–
Hou et al. (2010)^58^	Prevalence	Emotional	757	Medium	–	–
Hu et al. (2005)^39^	Prevalence	Emotional, physical	336	Medium	–	–
Huang et al. (2006)^36^	Prevalence	Emotional, physical, neglect	335	Medium	–	–
Lau et al. (2003)^88^	Consequences	Physical	489	Low	–	Physical – current smoker
						Physical – self-harm
Lau et al. (2005)^89^	Consequences	Physical	95 788	Low	–	Physical – current smoker
						Physical – illicit drug use
Li et al. (2012)^90^	Consequences	Sexual	4 084	Low	–	Sexual – depression
						Sexual – anxiety
Li et al.^a^ (2014)^60^	Prevalence	Emotional, physical	485	Low	–	–
Lin et al. (2011)^42^	Prevalence	Emotional, physical	7 475	Low	–	–
Lin et al. (2011)^76^	Prevalence	Sexual	683	Low	–	–
Lu et al. (2012)^24^	Prevalence	Emotional, physical	796	Medium	Emotional	–
Ma & Chen (2007)^70^	Prevalence and consequences	Physical	709	Medium	–	Physical– mental disorder
						Physical – depression
						Physical – anxiety
						Physical – current smoker
						Physical – problem drinking
Ma et al. (2005)^11^	Prevalence and consequences	Physical	528	Medium	–	Physical – mental disorder
						Physical – depression
						Physical – anxiety
Ma et al. (2012)^28^	Prevalence	Emotional, physical, neglect	475	Low	Neglect	–
Qiu & Ma (2010)^25^	Prevalence	Physical, sexual	709	Medium	Physical	–
Samuda (1988)^62^	Prevalence	Physical	100	Medium	–	–
Shen (2009)^69^	Prevalence	Physical	1 924	Low	–	–
Su et al. (2008)^81^	Prevalence and consequences	Sexual	1 386	Medium	–	Sexual – depression
						Sexual – anxiety
						Sexual – self-harm
Sun et al. (2006)^82^	Prevalence	Sexual	701	Medium	–	–
Tang (2002)^10^	Prevalence	Sexual	2 147	Medium	–	–
Tang et al. (2011)^91^	Consequences	Physical	6 564	Low	–	Physical – current smoker
						Physical – self-harm
Tao et al. (2006)^57^	Prevalence and consequences	Emotional, physical	5 141	Medium	–	Physical – mental disorder
						Emotional – mental disorder
Wang & Chen (2012)^40^	Prevalence	Emotional, physical	1 762	Medium	–	–
Xiao et al. (2008)^72^	Prevalence and consequences	Physical	10 894	Medium	–	Physical – self-harm
						Emotional – self-harm
Xiao (2008)^59^	Prevalence	Emotional, physical, neglect	2 073	Low	–	–
Xie et al. (2008)^27^	Prevalence	Emotional, neglect	457	Low	Neglect	–
Yan et al. (2009)^51^	Prevalence	Emotional, physical	1 200	Low	–	–
Yang et al. (2004)^23^	Prevalence	Emotional, physical, neglect	282	Medium	Emotional, physical, neglect	–
Yang (2012)^54^	Prevalence	Emotional, neglect	324	Low	–	–
Ye et al. (2006)^63^	Prevalence	Physical, sexual	5 141	Medium	–	–
Yen et al. (2008)^64^	Prevalence	Physical	1 684	Low	–	–
Yong et al. (2011)^52^	Prevalence	Emotional, physical	1 417	Low	–	–
Zeng et al. (2010)^44^	Prevalence	Emotional, physical, neglect	667	Low	–	–
Zhang et al. (2010)^74^	Prevalence	Neglect	3 539	Low	–	–
Zhao & Li (2006)^38^	Prevalence	Emotional, physical	485	Medium	–	–
Zhao et al. (2004)^47^	Prevalence	Emotional, physical, neglect	435	Low	–	–
Zhong et al. (2012)^41^	Prevalence	Emotional, physical	456	Medium	–	–
Zhou et al. (2010)^50^	Prevalence	Emotional, neglect	397	Low	–	–
Zhu et al. (2010)^49^	Prevalence	Emotional, neglect	659	Low	–	–
Zhu et al. (2012)^53^	Prevalence	Emotional, physical, neglect	2 374	Low	–	–

RR: relative risk.

^a^ This study was available online 22 October 2013 and therefore included in our systematic review.

Three studies were excluded from the final meta-analyses for each of the three types of child maltreatment: emotional abuse,^23^^,^^24^^,^^26^ physical abuse,^22^^,^^23^^,^^25^ and neglect.^23^^,^^27^^,^^28^ In these studies, subtraction of the unstandardized coefficients from the reported prevalence estimates produced negative values. Thus, the final five meta-analyses were based on 28 studies on emotional abuse, 33 on physical abuse, 15 on neglect and 16 on sexual abuse for females and 12 studies of sexual abuse of males (Table 2).

**Fig. 2:**
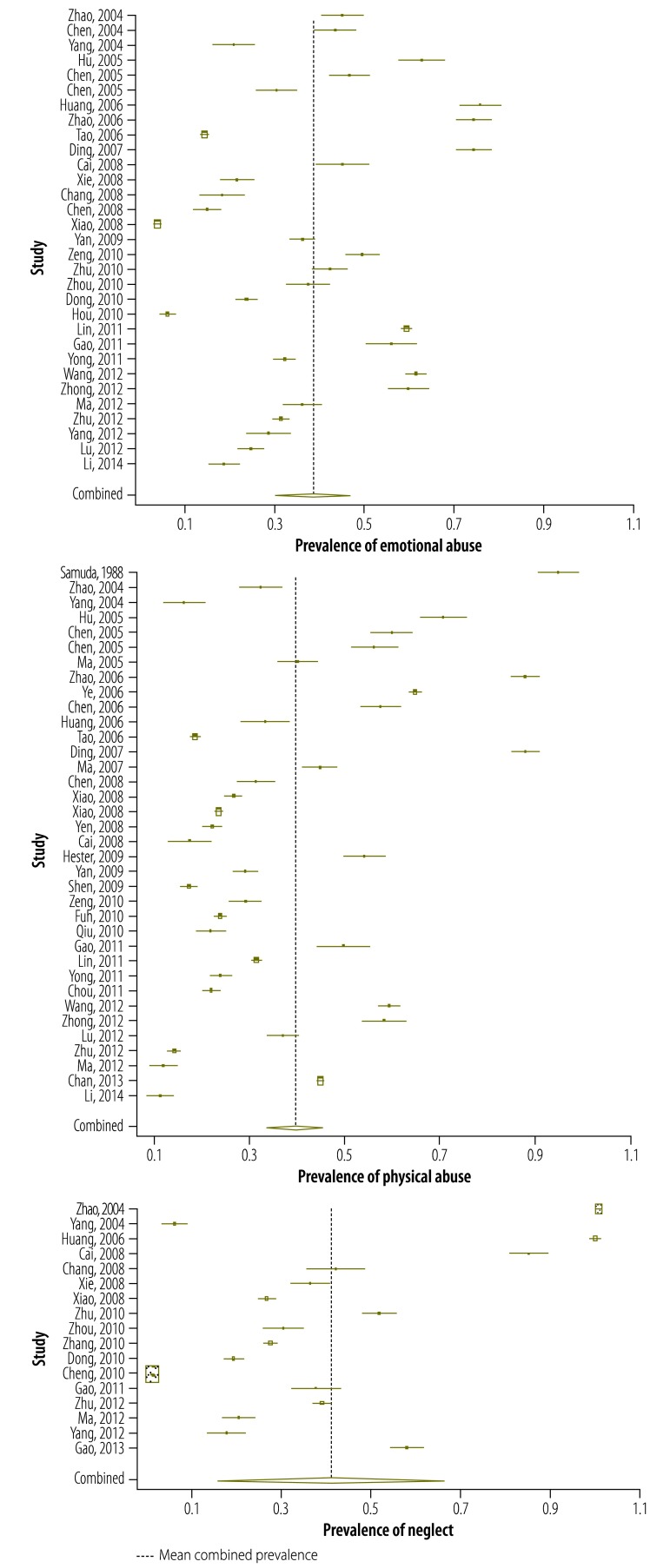
Studies reporting unadjusted prevalence for childhood emotional abuse, physical abuse and neglect, China, 1988–2013

**Fig. 3:**
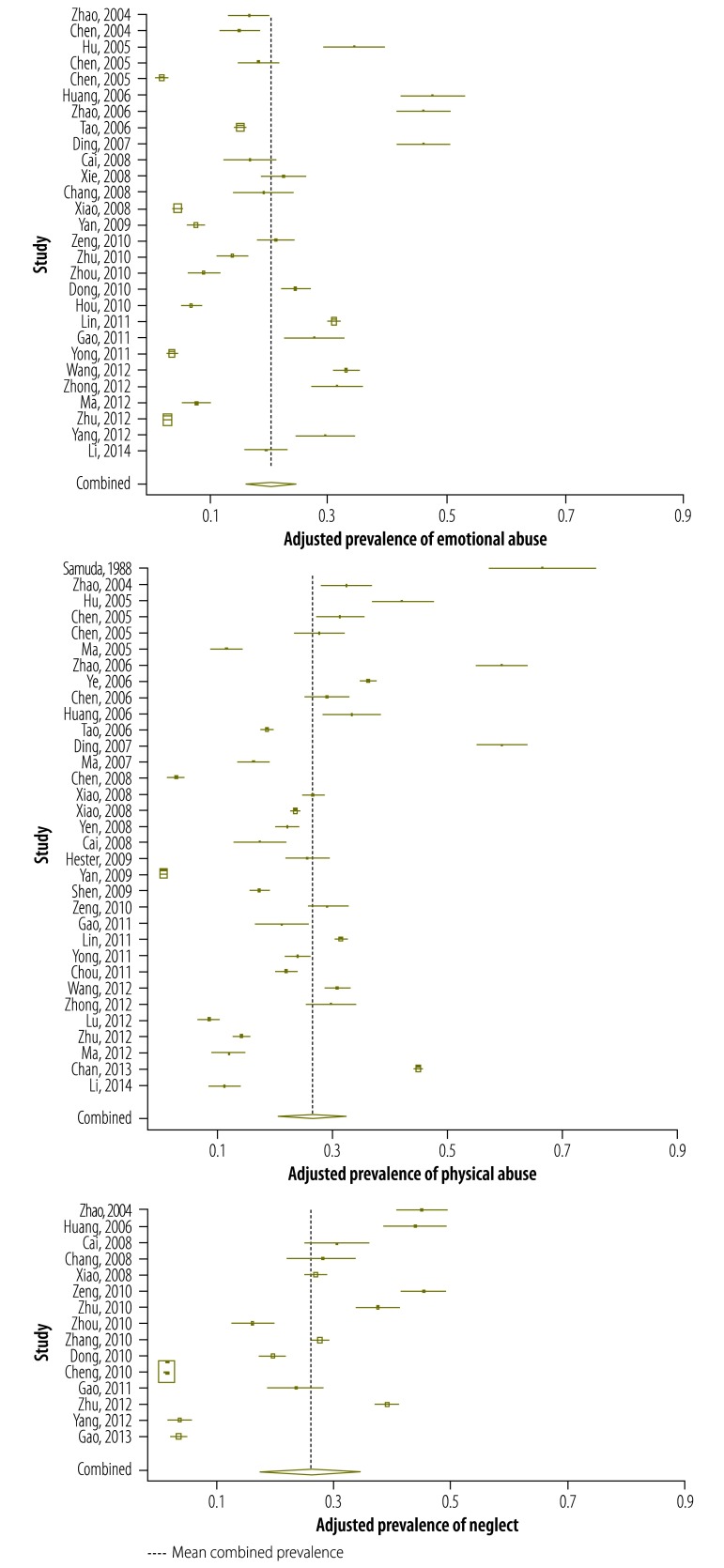
Adjusted prevalence of childhood emotional abuse, physical abuse and neglect, China, 1988–2013

The unadjusted and adjusted prevalence estimates from the included studies for emotional abuse, physical abuse and neglect are shown in Fig. 2 and Fig. 3. The estimates for sexual abuse have been published.^16^ Table 3 presents our unadjusted and adjusted estimates of the prevalence of each type of child maltreatment in China. Table 1 shows the RRs and population attributable fractions for the health and behavioural outcomes associated with each type of child maltreatment. No relevant data were available for child neglect. We estimate that for mental disorder, the population attributable fraction of emotional abuse is 26.3%, while the population attributable fraction of physical abuse is 18.8%. The population attributable fractions for physical abuse varied between 9.6% and 20.7% in the seven outcomes that we investigated. In general, the population attributable fractions for physical abuse were higher than those for sexual or emotional abuse.

**Table 3:** Estimated prevalence of four types of child maltreatment, China 1988–2013

Type of maltreatment	Unadjusted				Adjusted		
	Prevalence, % (95% CI)	No. of studies	Heterogeneity, *Q*		Prevalence, % (95% CI)	No. of studies	Heterogeneity, *Q*
**Physical abuse**	39.6 (33.7–45.5)	36	12 000^a^		26.6 (20.6–32.5)^b^	33	15 000^a^
**Emotional abuse**	38.6 (30.2–46.9)	31	11 000^a^		19.6 (15.4–23.7)^c^	28	4 556^a^
**Sexual abuse^d^**	8.7				–^e^	–	–
Females	9.5 (7.5–11.5)	16	414^a^		–	–	–
Males	8.0 (6.5–9.6)	12	149^a^		–	–	–
**Neglect**	41.2 (15.9–66.4)	18	73 000		26.0 (17.4–34.6)^f^	15	4 362^a^

CI: confidence interval.

^a^ *P* < 0.001.

^b^ Adjusted for use of a validated instrument.

^c^ Adjusted for repeated versus any abuse.

^d^ For sexual abuse, a meta-analysis was performed separately for boys and girls. The separate rates for boys and girls were then combined to obtain an overall rate for sexual abuse, using the sex proportions as weights.

^e^ Not adjusted because no methodological factors significantly predicted the prevalence of sexual abuse.

^f^ Adjusted for whether a study asked specific questions and for sample size.

The numbers and economic values of the DALYs lost because of child maltreatment are shown in Table 4. Although only a limited number of health outcomes were considered, an estimated 11 288 100 of DALYs lost in China in 2010 were attributable to child physical abuse. The estimated economic value of these lost DALYs was 50 billion United States dollars – or 0.84% of China’s gross domestic product in 2010. Even though we only considered the impacts of child emotional abuse on mental health disorders and self-harm, we estimated that such abuse caused 6 334 700 of the DALYs lost in China in 2010. The DALYs lost in 2010 because of child emotional and sexual abuse had estimated values equivalent to 0.47% and 0.39% of China’s gross domestic product in 2010, respectively.

**Table 4:** Estimates of the disability-adjusted life-years and economic value lost because of child abuse, China, 2010

Outcome of maltreatment	Physical abuse				Emotional abuse				Sexual abuse		
	DALYs lost (x 1000)	Value lost			DALYs lost (x 1000)	Value lost			DALYs lost (x 1000)	Value lost	
		Millions of US$	% of GDP			Millions of US$	% of GDP			Millions of US$	% of GDP
**Mental disorder**	3 924.5	17 399.1	0.29		5 490.8	24 342.7	0.41		NA	NA	NA
Depression	1 429.9	6 339.2	0.11		NA	NA	NA		639.0	2 832.9	0.05
Anxiety	490.5	2 174.4	0.04		NA	NA	NA		127.2	563.8	0.01
**Current smoker**	2 885.5	12 792.6	0.22		NA	NA	NA		2 577.1	11 425.4	0.19
**Problem drinking**	2 849.4	12 632.5	0.21		NA	NA	NA		1 173.5	5 202.8	0.09
**Illicit drug use**	538.4	2 387.0	0.04		NA	NA	NA		NA	NA	NA
**Self-harm**	1 090.3	4 833.7	0.08		843.9	3 741.5	0.06		644.1	2 855.4	0.05
**Total**	**11 288.1^a^**	**50** **045.0^a^**	**0.84^a^**		**6** **334.7**	**28 084.2**	**0.47**		**5** **160.9**	**22 880.3**	**0.39**

DALYs: disability-adjusted life-years; GDP: gross domestic product; NA: not available; US$: United States dollars.

^a^ Depression and anxiety are included in mental disorder, therefore they do not contribute to the total value.

Note: Inconsistencies arise in some values due to rounding.

## Discussion

We estimated the general burden of child maltreatment in China. Maltreatment is a common experience for Chinese children. Despite a paucity of data on the impact of child maltreatment on child and adult health, the associations between such maltreatment and subsequent poor mental health and harmful behaviours in China are substantial and consistent with the results of research elsewhere.^92^^,^^93^ According to our calculations, 11.3 million of the DALYs lost in China in 2010 were attributable to child physical abuse. This value lies between the corresponding estimates for diabetes mellitus – 7.8 million DALYs lost – and ischaemic heart disease – 17.8 million DALYs lost.^30^ The size of this burden justifies further research and increased efforts to improve child protection in China, especially since our estimates of the burdens of child maltreatment are based on the available data on a small number of health outcomes and are therefore likely to be underestimates.

There is a paucity of Chinese data on child neglect and emotional abuse and their associated consequences. If the financial burden of child maltreatment is to be accurately assessed in China, there is also a need for additional information on child-maltreatment-attributable losses in productivity and the short- and long-term medical costs of child maltreatment.^5^ Another considerable gap in our current understanding is that, as no community-based study on temporal changes in child maltreatment in China has been published, it remains unclear if the problem is getting better or worse or staying unchanged. Population-based research that provides estimates of the temporal changes across a broad spectrum of childhood abuse, neglect and other adversities should be a core element of any comprehensive national prevention response.

Our study had several limitations and had several major gaps in the relevant evidence base. Most of the data that we used for calculating prevalence and population attributable fractions came from studies that did not employ representative samples. Many of our included studies only investigated one type of maltreatment or – if they investigated several types – did not report disaggregated data. The high level of variation in the reported prevalence of maltreatment is worrying and possibly indicative of substantial variation in how maltreatment has been defined and evaluated and in the sampling method used. Population attributable fractions can be sensitive to small changes in prevalence and RR and this problem may be exacerbated when the fractions are based on data from multiple studies. Although we carefully reviewed all input data to select appropriate studies, our results rest squarely on the – often uncertain – quality of the available data. By using DALYs, we were only able to estimate the non-fatal health burden posed by child maltreatment. We could find no data on maltreatment-attributable child mortality in China. However, WHO recently estimated that, in China, 1266 children aged 0–14 years died from interpersonal violence in 2012 – resulting in 111 170 years of life lost.^94^ It seems very likely that, in China, there are also violent deaths among adolescents aged 15–18 years and that some children commit suicide as a result of maltreatment. 

As some of the health outcomes that we investigated may have overlapped, our estimates may have been affected by the double-counting of DALYs lost. However, we carefully scrutinized all study inputs and population attributable fractions to try to minimize this problem. As far as possible, we maintained one-to-one correspondence between the population attributable fractions and the burden measures from the global burden of disease 2010 study in China.

Many of the studies that we included in our review excluded many possibly important confounding factors and may therefore have overestimated the direct effects of child maltreatment. For example, it is almost impossible to know if genetic inheritance may explain some portion of the associations between maltreatment and outcomes. The accuracy of our estimates was also limited by the fact that most of the data on prevalence and outcomes that we used were self-reported in cross-sectional studies where maltreatment was measured retrospectively.

After considering all of the limitations of our study, we think that our burden estimates are probably underestimates of the true values. Many of the serious effects of child maltreatment – e.g. poor educational and employment outcomes, high levels of health-care utilization, criminal behaviour and reproductive health problems – were not included because no relevant studies have been published. In addition, no estimates are available of the costs to the Chinese police and child welfare services of child maltreatment.

Despite the gaps in the current evidence base, this study indicates the importance of prioritizing child maltreatment as a key health concern in China. It also underscores the need to steer resources towards child protection and to strengthening the knowledge base regarding the scale and consequences of child maltreatment at national level.
